# An Ash2L/RbBP5 Heterodimer Stimulates the MLL1 Methyltransferase Activity through Coordinated Substrate Interactions with the MLL1 SET Domain

**DOI:** 10.1371/journal.pone.0014102

**Published:** 2010-11-23

**Authors:** Fang Cao, Yong Chen, Tomasz Cierpicki, Yifan Liu, Venkatesha Basrur, Ming Lei, Yali Dou

**Affiliations:** 1 Department of Pathology, University of Michigan, Ann Arbor, Michigan, United States of America; 2 Department of Biological Chemistry, University of Michigan, Ann Arbor, Michigan, United States of America; 3 Howard Hughes Medical Institute, University of Michigan, Ann Arbor, Michigan, United States of America; Texas A&M University, United States of America

## Abstract

Histone H3 lysine 4 (K4) methylation is a prevalent mark associated with transcription activation and is mainly catalyzed by the MLL/SET1 family histone methyltransferases. A common feature of the mammalian MLL/SET1 complexes is the presence of three core components (RbBP5, Ash2L and WDR5) and a catalytic subunit containing a SET domain. Unlike most other histone lysine methyltransferases, all four proteins are required for efficient H3 K4 methylation. Despite extensive efforts, mechanisms for how three core components regulate MLL/SET1 methyltransferase activity remain elusive. Here we show that a heterodimer of Ash2L and RbBP5 has intrinsic histone methyltransferase activity. This activity requires the highly conserved SPRY domain of Ash2L and a short peptide of RbBP5. We demonstrate that both Ash2L and the MLL1 SET domain are capable of binding to S-adenosyl-L- [methyl-^3^H] methionine in the MLL1 core complex. Mutations in the MLL1 SET domain that fail to support overall H3 K4 methylation also compromise SAM binding by Ash2L. Taken together, our results show that the Ash2L/RbBP5 heterodimer plays a critical role in the overall catalysis of MLL1 mediated H3 K4 methylation. The results we describe here provide mechanistic insights for unique regulation of the MLL1 methyltransferase activity. It suggests that both Ash2L/RbBP5 and the MLL1 SET domain make direct contacts with the substrates and contribute to the formation of a joint catalytic center. Given the shared core configuration among all MLL/SET1 family HMTs, it will be interesting to test whether the mechanism we describe here can be generalized to other MLL/SET1 family members in the future.

## Introduction

MLL1 (mixed lineage leukemia) is a mammalian homolog of the *Drosophila* Trithorax protein (Trx). It is essential for definitive hematopoiesis by regulating transcription activation of *Hox* genes (e.g. *Hoxa9* and *Meis1*), which encode transcription/regulatory factors promoting hematopoetic stem cell expansion. MLL1 is an H3 K4 histone methyltransferase (HMT) that effect mono-, di-, and tri-methylation through its evolutionarily conserved SET (Su (var), Enhancer of Zeste and Trithorax) domain [1], [2], [3]. Both MLL1 and H3 K4 methylation are localized broadly across promoter, 5′ transcribed and coding regions of the critical target genes and facilitate the recruitment of RNA Pol-II and other chromatin remodeling activities involved in transcription activation [4], [5], [6]. Deregulation of MLL1 is associated with acute lymphoid and myeloid leukemia [7], [8], [9]. In most cases, balanced chromosome translocations occur on one MLL1 allele and result in leukemogenic MLL1 fusion proteins (e.g. MLL1-AF9, MLL1-ENL) [7], [8]. MLL1 fusion proteins, which lack the C-terminal SET domain, cooperate with the remaining copy of wild type MLL1 in leukemogenesis [10], [11]. Therefore, understanding the regulation of MLL1 methyltransferase activity bears clinical significance in the treatment of MLL1 deregulated leukemia.

MLL1 is one of the MLL/SET1 family HMTs for H3 K4 methylation in higher eukaryotes. MLL/SET1 family HMTs include SET1A and SET1B [12], the mammalian orthologues of ySET1, and four MLLs (MLL1-4), which share limited homology with ySET1 beyond the SET domain [1], [3], [13], [14], [15]. Biochemical studies have shown that MLL/SET1 family histone methyltransferases share a core configuration that includes three common proteins: RbBP5, Ash2L and WDR5 [1], [3], [13], [14], [15]. The presence of such a structural core in MLL/SET1 family HMTs implies common regulatory mechanisms for their enzymatic activities and/or substrate specificities. Indeed, unlike most histone lysine methyltransferases, optimal methyltransferase activity for MLL1 depends on its interaction with all three proteins [16]. Using *in vitro* biochemical reconstitution, we have demonstrated that a four-component MLL1 core complex is sufficient to recapitulate most of the methyltransferase activity of the MLL1 holo-complex, which is significantly higher than MLL1 alone [16]. Later biochemical studies have confirmed and further explored the detailed structural organization of the MLL1 core complex [17], [18]. In particular, it has been reported that WDR5 is important in maintaining the integrity of the MLL1 complex by directly interacting with MLL1 through a conserved Arginine (R) residue in the MLL1 pre-SET domain [17], [19], [20] and with RbBP5 [21]. However, WDR5 by itself does not stimulate methyltransferase activity of the MLL1 SET domain *in vitro* [17], [20], [22]. In contrast, RbBP5 and Ash2L contribute significantly to the overall activity of the MLL1 complex despite their lack of stable association with the MLL1 SET domain in the absence of WDR5 [16], [22]. The mechanism for RbBP5 and Ash2L mediated stimulation of MLL1 activity remains elusive and is the focus of this study.

With the exception of Dot1, most histone lysine methyltransferases contain the evolutionarily conserved SET domain [23]. Biochemical and structural studies revealed that SET domains usually adopt structures featuring a narrow hydrophobic channel that links the substrate lysine and S-adenosyl-L- [methyl-^3^H] methionine (SAM) [24]. This lysine access channel, formed by conserved aromatic residues, is essential for optimal alignment between the methyl-group of SAM and the ε-amine group of the lysine residue required for efficient catalysis [23], [24]. It is also important for determination of the methylation state specificity [24]. For most lysine methyltransferases, SET domains alone are sufficient to carry out the methyl-transfer reaction. Notable exceptions to the rule are the MLL/SET1 family H3 K4 methyltransferases and the Polycomb group protein EZH2, which are fully active only in the context of the complexes [16], [25], [26]. Recent report of the co-crystal structure of the MLL1 SET domain in complex with AdoHcy and an H3 peptide suggests that the lysine access channel of MLL1 SET may be widely exposed in the native protein, rendering it inefficient in aligning the ε-amine of substrate lysine with SAM for optimal catalysis [22]. The unique structure of the MLL1 SET domain and the requirement of other proteins for optimal methyltransferase activity support a hypothesis that other members of the MLL1 complex contribute to the proper alignment of the substrate lysine with SAM [22].

Recently, a SET domain independent methyltransferase activity has been reported for the MLL1 core complex [18]. This activity mainly targets H3 K4 mono-methylation [18]. Here we show that non-SET proteins RbBP5 and Ash2L, acting as a heterodimer (referred to as Ash2L/RbBP5 herein), account for this novel methyltransferase activity. By comprehensive mapping of both RbBP5 and Ash2L, we found that the Ash2L SPRY domain and a short RbBP5 peptide are important for both their intrinsic activity and the overall activity of the MLL1 complex. Importantly, Ash2L/RbBP5 is able to directly interact with substrates H3 and SAM and contributes significantly to the overall catalysis of the MLL1 complex. Our results support a model that Ash2L/RbBP5, just like the MLL1 SET domain, directly participates in the catalytic reaction of H3 K4 methylation in the MLL1 complex. Our study presents a new paradigm for the regulation of histone lysine methyltransferases.

## Results

### Ash2L/RbBP5 stimulate MLL1 activity in the absence of WDR5

**Figure 1:**
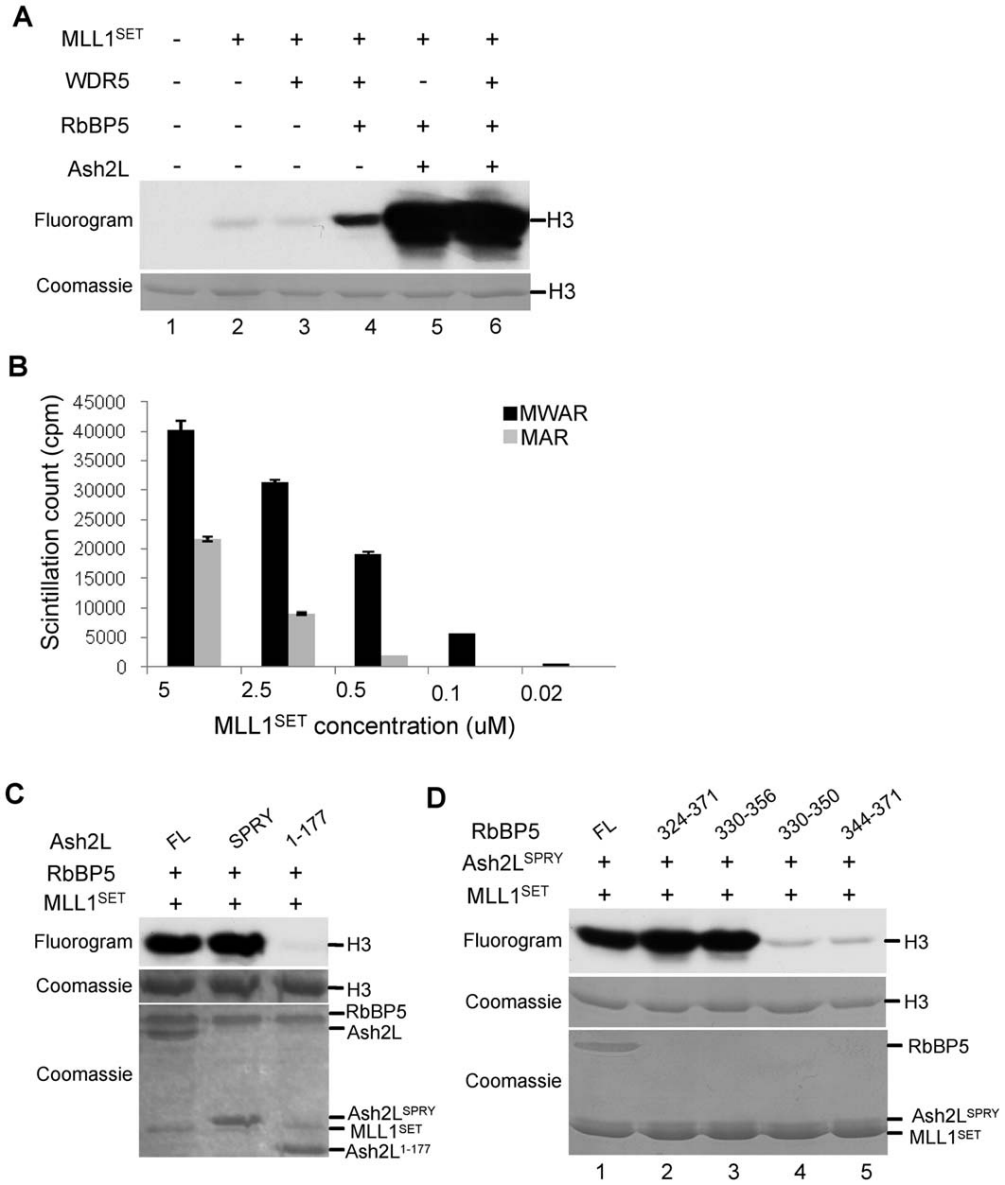
Ash2L/RbBP5 stimulates MLL1 activity in the absence of WDR5. (**A**) *In vitro* HMT assay for MLL1^SET^ and MLL1^SET^ with one, two or three components of the MLL1 complex as indicated on top. For all reactions, 5 µM of MLL1^SET^ was used and all other MLL1 core components were added at equal molar concentration. (**B**) Scintillation count for *in vitro* HMT assays using either four-component complex MWAR or three-component mixture MAR as enzymes. Y-axis, scintillation counts (cpm) for the methylation reaction. X-axis, molar concentration of MLL1^SET^ used in different reactions. Other proteins were added at equal molar concentration to MLL1^SET^ in each reaction. All experiments were repeated for three times. The error bar represented standard deviation. (**C**) *In vitro* HMT assay using various Ash2L fragments as indicated on top. Top panel, fluorogram of methylated H3. Bottom two panels, Coomassie stained gels for H3 substrate and the MLL1 core components used in the same reaction. (**D**) *In vitro* HMT assay using various RbBP5 peptides at equal molar concentration (5 µM) as indicated on top. Top panel, fluorogram of methylated H3. Bottom two panels, Coomassie stained gels for H3 substrate and the MLL1 core components used in the same reaction. Ash2L^SPRY^ and MLL1^SET^ were run at about the same position. RbBP5 peptides were run out of the gel due to their small sizes.

In order to study the function of RbBP5 and Ash2L in the MLL1 complex, we expressed WDR5 (W, Δ23aa), RbBP5 (R), Ash2L (A, isoform b, 534aa) and the MLL1 SET domain (MLL1^SET^ or M, residues 3754-3969) in *E. coli* and purified them to homogeneity respectively (Fig. S1A). The purity of the recombinant proteins was further confirmed by mass spectrometry (data not shown). Different combinations of these four proteins were mixed together and tested for HMT activity *in vitro*. Consistent with previous studies [16], [22], dramatic increase in H3 methylation was observed when stoichiometric quantities of four core components were present (Fig. 1A, lane 6 vs 2). Surprisingly, RbBP5 and Ash2L were able to stimulate the MLL1^SET^ activity to the same level as the four-component complex (MWAR) at 5 µM concentration for each protein (Fig. 1A, lane 5 vs 6). In contrast, WDR5, which makes direct contact with MLL1 through a “WIN” motif [19], [20], could not stimulate MLL1 activity at the same assay condition *in vitro* (Fig. 1A, lane 3 vs lane 2). Given the essential role of WDR5 in maintaining the integrity of the MLL1 complex and in H3 K4 methylation *in vivo* [16], [27], we further compared the methyltransferase activity of the four-component complex with that of the three-component mixture (MAR) at different complex concentrations. As shown in Fig. 1B, we found that the requirement of WDR5 in overall methyltransferase activity was sensitive to complex concentration (Fig. 1B). When complex concentration was low (e.g. 0.5 µM), there was ∼10-fold difference in activities of MWAR versus MAR (Fig. 1B). In contrast, when complex concentration was high (e.g. 5 µM), the difference was reduced to ∼2 fold. Given that neither RbBP5 nor Ash2L stably interacts with MLL1^SET^ in the absence of WDR5 [16], [22] and that their stimulatory effects to MLL1^SET^ activity are more apparent at high complex concentration, it is likely that some previously uncharacterized transient interactions between Ash2L/RbBP5 and MLL1^SET^ are important for catalyzing H3 K4 methylation.

### RbBP5 and Ash2L function as a heterodimer

In order to study the underlying mechanism for Ash2L/RbBP5 mediated stimulation of MLL1^SET^ activity, we first mapped Ash2L and RbBP5 for minimal domains that support the overall activity of the MLL1 core complex. As shown in Fig. 1, Ash2L C-terminal SPRY domain (Fig. 1C) and a short RbBP5 peptide (330-356aa) (Fig. 1D) were sufficient to stimulate the activity of MLL1^SET^. For all reactions in Fig. 1C and 1D, equal molar amount of proteins or peptides were used. RbBP5 peptides were run out of the gel due to their small sizes (Fig. 1D). The Ash2L SPRY domain is evolutionarily conserved, with ∼50% sequence identity/similarity among Ash2L homologues in *Homo sapiens*, *Drosophila, S. cerevisiae* and *Tetrahymena thermophila* (Fig. S2). It has been proposed that the SPRY domain is a protein-protein interaction domain [28], [29]. Unlike Ash2L, the required RbBP5 peptide (330-356aa) is highly acidic, with 30% residues being glutamic acid (E) or aspartic acid (D). By the thermal shift assay [30], this RbBP5 peptide (330-356aa) did not show any thermal transition, suggesting no secondary structure in solution (Fig. S3). Interestingly, it includes residues 350–356 that are essential for Ash2L interaction [16]. Deleting this Ash2L interacting region in RbBP5 abolished the Ash2L/RbBP5 dependent stimulation of the MLL1^SET^ activity (Fig. 1D, lane 4). This result suggests that Ash2L and RbBP5 probably function together as a heterodimer in the complex. This is consistent with the observation that neither RbBP5 nor Ash2L alone was able to stimulate MLL1^SET^ activity to the same extent as when they were together (Fig. 1A and data not shown).

### Ash2L/RbBP5 has intrinsic methyltransferase activity

**Figure 2:**
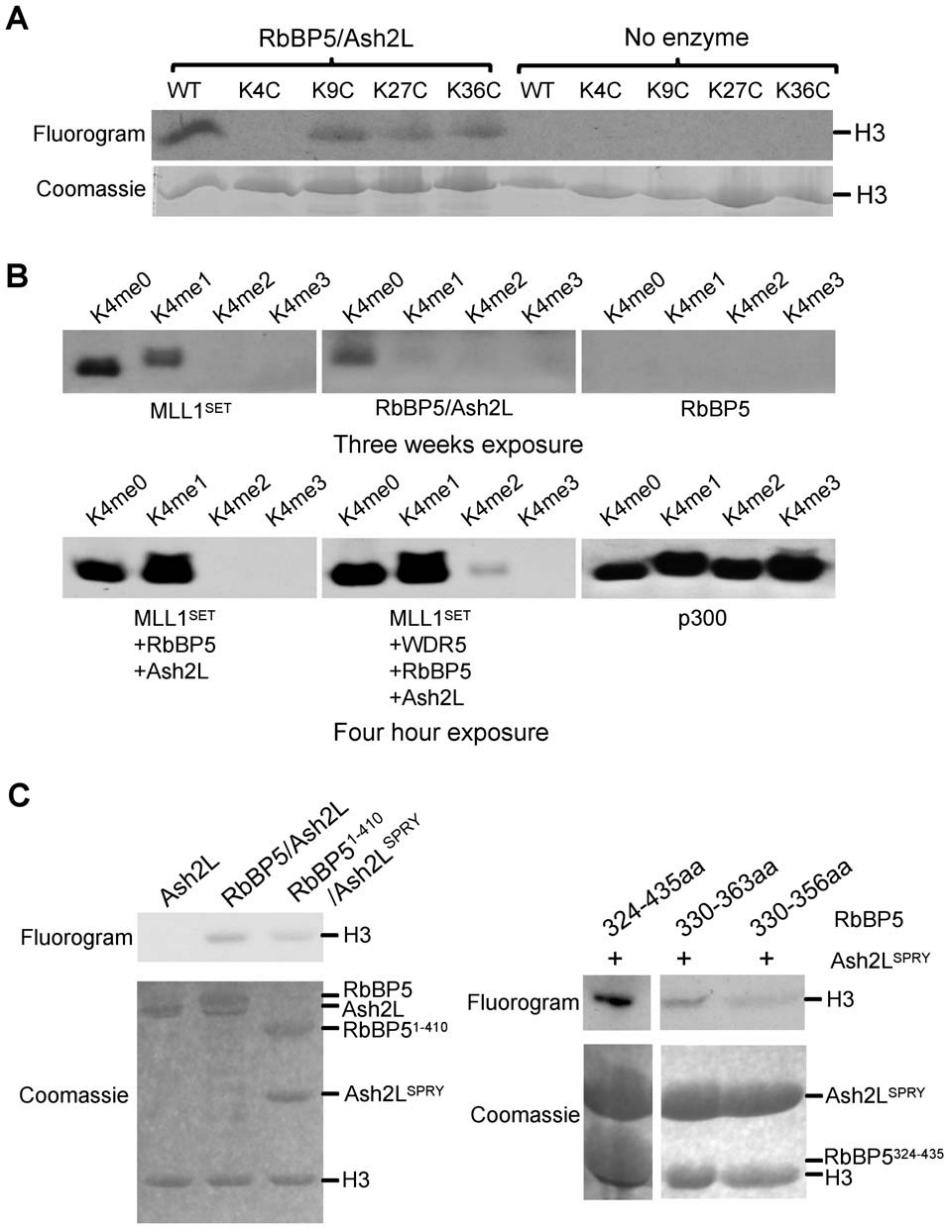
Ash2L/RbBP5 has intrinsic methyltransferase activity. (**A**) *In vitro* HMT assay using ∼5 µM Ash2L/RbBP5 heterodimer as enzymes. Top, fluorogram of methylated H3 after three weeks of exposure. Bottom, Coomassie stained gels for H3 substrate. (**B**) *In vitro* HMT assay using 60 µM unmodified, mono-, di- and tri-methylated H3 K4 peptides as substrates. The proteins (∼5 µM final concentration) in each reaction were indicated on bottom. *In vitro* HAT assay using p300 was included as a quality and loading control for H3 peptides. The exposure times for the top and bottom panels were different and were indicated on bottom. (**C**) Left**,** *in vitro* HMT assay for Ash2L, Ash2L/RbBP5 as well as Ash2L^SPRY^/RbBP5^1-410^. Right, *in vitro* HMT assay for Ash2L^SPRY^ and various RbBP5 peptides as indicated on top. Coomassie stained gels for H3 and proteins used in the assays were included on bottom.

Given the reported presence of a SET domain independent methyltransferase activity in the MLL1 core complex [18], we tested whether RbBP5 and Ash2L possess intrinsic histone methyltransferase activity. Using *in vitro* HMT assays, we found that Ash2L/RbBP5 exhibited intrinsic HMT activity for histone H3 (Fig. 2A). As shown in Fig. 2A, Ash2L/RbBP5 demonstrated specificity mainly towards H3 K4 (Fig. 2A). Mutation of K4, but not other lysine residues, abolished H3 methylation by Ash2L/RbBP5. When H3 peptides carrying one, two or three methyl-groups at K4 were used as the substrates, Ash2L/RbBP5 was more efficient in methylating unmodified H3 peptides (Fig. 2B). In contrast to both MLL1^SET^ and the MLL1 core complex, activity of Ash2L/RbBP5 on H3 peptides with mono- or di-methylated K4 was minimal (Fig. 2B). H3 peptide with tri-methylated K4 was used as the negative control (Fig. 2B). This result was reminiscent of what was described previously for the SET-independent activity of the WAR complex [18]. Of note, the activity we observed for Ash2L/RbBP5 was much weaker compared to MLL1^SET^ and required long exposure for detection (Fig. 2B). Further characterization showed that the same domains in Ash2L and RbBP5 that were important for overall H3 methylation also supported the intrinsic methyltransferase activity. As shown in Fig. 2C, the Ash2L SPRY domain and RbBP5 330-356aa were capable of methylating recombinant histone H3 in the *in vitro* HMT assays even though they were less active compared to full-length proteins. The weaker activities of Ash2L SPRY domain and RbBP5 shorter peptides suggest that other sequences in Ash2L or RbBP5 may also be important for the intrinsic activity of the Ash2L/RbBP5 heterodimer. In addition, neither Ash2L nor RbBP5 alone had any detectable HMT activity under the same assay condition (Fig. 2B and 2C).

### Ash2L is capable of binding to substrate SAM

**Figure 3:**
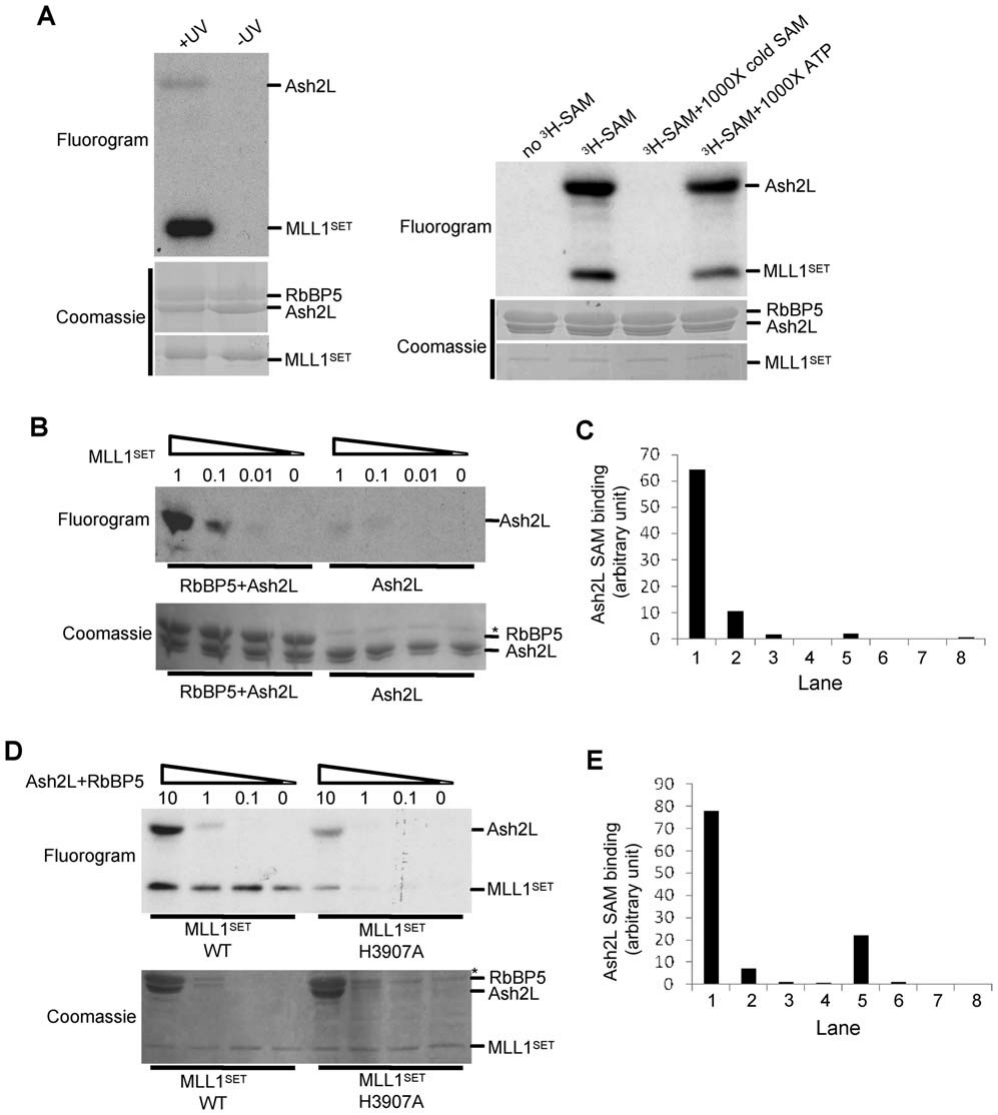
Ash2L/RbBP5 binds to substrate SAM. (**A**) SAM binding assays for Ash2L/RbBP5 and MLL1^SET^ by UV cross-linking. Left, SAM binding assays for the MLL1 core complex with or without UV cross-linking. Equal molar (∼3 µM) of each MLL1 core component was used. Right, SAM binding by Ash2L and MLL1^SET^ could be chased off by 1000x excess cold SAM (∼0.35 mM) but not by ATP (∼0.35 mM). Black lines indicated expected positions for each protein. 10-fold molar excess of Ash2L/RbBP5 (relative to MLL1^SET^) was used in this experiment to reduce the exposure time for detecting Ash2L SAM binding. Black lines indicated expected positions for each protein. (**B**) SAM binding assay for 3 µM equivalent of Ash2L alone or Ash2L/RbBP5 in the presence of increasing amount of MLL1^SET^. Molar ratios of MLL1^SET^ versus Ash2L/RbBP5 or Ash2L were indicated on top. (**C**) Image-J quantitation of the results in (B). Y-axis, arbitrary unit for pixel count. (**D**) Reciprocal SAM binding assays as performed in (B) except ∼3 µM wild type or H3907A MLL1^SET^ was mixed with increasing amount of Ash2L/RbBP5. The molar ratios of Ash2L/RbBP5 versus MLL1^SET^ were indicated on top. (**E**) Image-J quantitation of the results in (D). Y-axis, arbitrary unit for pixel count. In (**B**) and (**D**), Coomassie staining for the same gels was included on bottom. The positions for Ash2L and RbBP5 were indicated on left. *, non-specific protein.

Given the intrinsic activity of the Ash2L/RbBP5 heterodimer, we next examined whether it bound to substrate SAM, an essential step in catalysis. We found that Ash2L was capable of binding to SAM in the presence of RbBP5 and MLL1^SET^ after UV treatment (Fig. 3A). In contrast, no SAM binding by RbBP5 was detected in the same reaction (Fig. 3A). SAM binding by Ash2L was UV-dependent and could be competed off by excess amount of unlabeled SAM (1000 fold, ∼0.35 mM) (Fig. 3A). As a control, excess amount of ATP (1000 fold, ∼0.35 mM) could not compete off the SAM binding by Ash2L or MLL1^SET^. Interestingly, stable Ash2L SAM binding required both RbBP5 and MLL1^SET^. As shown in Fig. 3B, SAM-binding by Ash2L was severely comprised when MLL1^SET^ and RbBP5 were either sub-stoichiometric or absent. Quantitation of Ash2L SAM binding in Fig. 3B was included in Fig. 3C. In contrast to Ash2L, MLL1^SET^-SAM interaction was stable and was not affected by Ash2L/RbBP5 (Fig. 3D). Adding excess amount of Ash2L/RbBP5 to wild type MLL1^SET^ did not enhance SAM binding by wild type MLL1^SET^. However, Ash2L/RbBP5 was able to partially rescue the SAM binding deficiency of the MLL1^SET^ mutant H3907A, which was deficient in SAM binding by itself (Fig. 3D) [3]. Quantitation of Ash2L SAM binding in Fig. 3D was included in Fig. 3E. These results suggest that two proteins in the MLL1 core complex (i.e. Ash2L and MLL1^SET^) are capable of SAM binding and their interactions with SAM can potentially be influenced by each other.

### Mutations that disrupt RbBP5 and Ash2L interaction abolish Ash2L SAM binding and H3 K4 methylation

**Figure 4:**
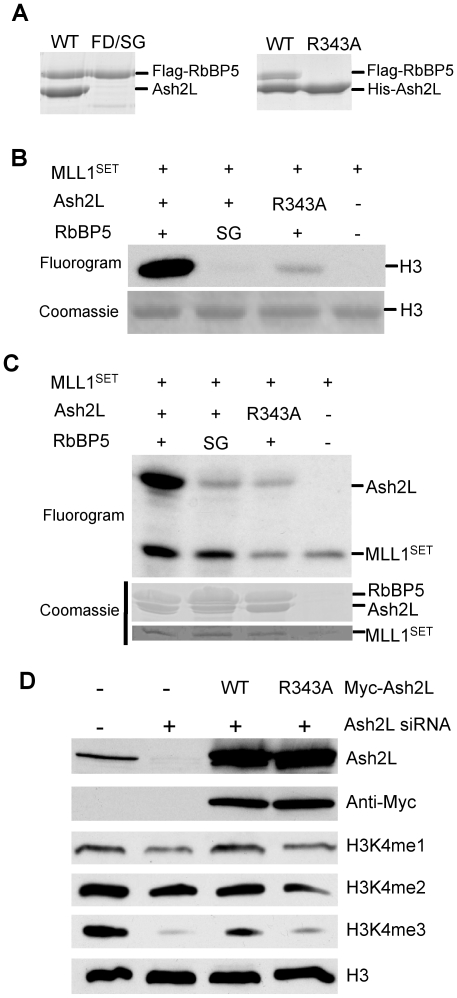
Ash2L/RbBP5 is important for the overall activity of the MLL1 complex. (**A**) Co-immunoprecipitation of RbBP5 and Ash2L. Proteins purified through either Flag tag on RbBP5 or RbBP5 mutant (left) or His tag on Ash2L or Ash2L mutant (right) were run on SDS-PAGE for Coomassie staining. (**B**) *In vitro* HMT assay for MLL1^SET^, Ash2L or Ash2L R343A mutant and RbBP5 or RbBP5 mutant as indicated on top. Equal amount of proteins and H3 substrate were used in each reaction. (**C**) SAM binding assays using proteins indicated on top. Positions of Ash2L and MLL1^SET^ were indicated on right. In (**B**)**-**(**C**), SG refers to RbBP5 F352S/D353G double mutations. Coomassie stained gels were included on bottom as controls. (**D**) HeLa cells were treated with siRNAs targeting endogenous Ash2L transcripts for 48hrs and then transfected with plasmids expressing Myc-tagged Ash2L or Ash2L mutant R343A. Total proteins were separated on SDS-PAGE and blotted for different antibodies indicated on right. Anti-H3 antibody was used as the loading control.

Given the importance of RbBP5 and Ash2L interaction in the overall H3 K4 methylation, we next tested whether disrupting RbBP5 and Ash2L interaction by site-directed mutagenesis reduced SAM binding by Ash2L. As previously demonstrated, the minimal Ash2L interacting region in RbBP5 (350-356aa) is highly acidic, with 30% residues being glutamic acid (E) or aspartic acid (D). We reason that the residues in Ash2L that are important for RbBP5 interaction may involve positively charged residues such as Lysine (K) or Arginine (R). By sequence alignment, we found that a couple of Arginine residues in Ash2L were highly conserved through evolution (square, Fig. S2). Mutating one of them, R343A, completely abolished Ash2L interaction with RbBP5 (Fig. 4A). Reciprocally, we also introduced mutations F352S/D353G to RbBP5 (RbBP5^SG)^, which was previously showed to disrupt Ash2L interaction [16](Fig. 4A). When RbBP5^SG^ or Ash2L^R343A^ mutant was used in the *in vitro* HMT assay, they failed to support overall H3 K4 methylation (Fig. 4B). Strikingly, both mutants led to reduction in Ash2L SAM binding as well even though SAM-binding by MLL1^SET^ was not affected (Fig. 4C). The SAM binding deficiency of Ash2L mutant was not due to protein mis-folding since mutated Ash2L and RbBP5 proteins have similar thermal stability as the wild type proteins in the thermal shift assay (Table S1) [30]. Taken together, these results argue that SAM binding by Ash2L probably plays a previously uncharacterized role in the regulation of the overall activity of the MLL1 complex.

To further confirm the function of Ash2L in mammalian cells, we tested the ability of Ash2L^R343A^ in rescuing global reduction of H3 K4 methylation after RNAi knockdown of endogenous Ash2L [16]. The siRNAs we used specifically targeted 3′UTR of Ash2L transcripts from the endogenous locus. As a result, Ash2L knockdown led to global down regulation of H3 K4 tri-methylation (Fig. 4D). H3 K4 mono-methylation was also slightly affected by Ash2L knockdown. Over-expression of Myc-tagged wild-type Ash2L in cells treated with Ash2L siRNA partially rescued the global reduction of H3 K4 tri-methylation. In contrast, over-expression of Ash2L^R343A^ could not rescue H3 K4 tri-methylation defects (Fig. 4D). Immunoblots for H3 and myc-Ash2L were included as loading and expression controls respectively.

### SAM binding by Ash2L, not the intrinsic activity of MLL1^SET^, correlates with overall activity of the MLL1 core complex

**Figure 5:**
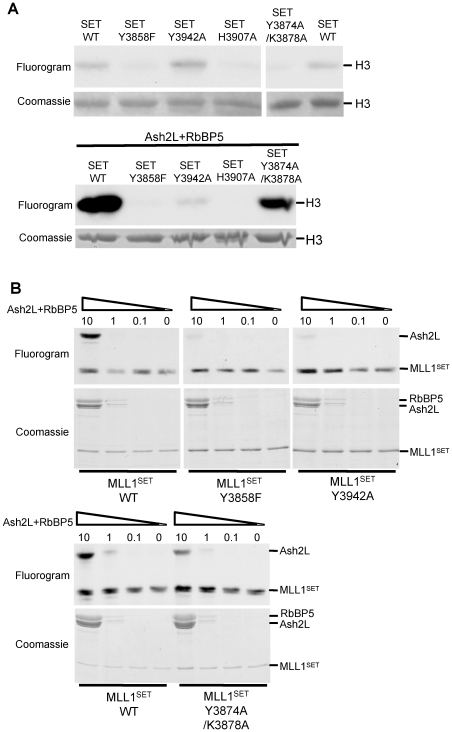
Ash2L/RbBP5 and MLL1^SET^ coordinate in H3 K4 methylation. (**A**) *In vitro* HMT assays using ∼5 µM of wild type and mutants MLL1^SET^ either alone (top) or with Ash2L/RbBP5 (bottom) as enzymes (**B**) SAM binding assays for Ash2L and either wild type MLL1^SET^ or SET domain mutants. 3 µM of wild type MLL1^SET^ or SET domain mutants was used in each reaction. Molar ratios of Ash2L/RbBP5 versus MLL1^SET^ were indicated on top. The positions for Ash2L and RbBP5 were indicated on left. Duplicate samples were run for Coomassie staining as the loading controls.

To further study the functions of Ash2L SAM binding, we next tested its function in the context of MLL1^SET^ mutants. There are several previously characterized MLL1^SET^ mutants including Y3858F, Y3942A and Y3874A/K3878A [22]. Both Y3858 and Y3942 are at the catalytic center of the MLL1 SET domain and Y3942 is important for the state specificity of MLL1^SET^ alone [18]. In contrast, Y3874 and K3878 are at the SET-I sub-domain away from the active center [22]. Consistent with previous observation [22], the intrinsic activity of these MLL1^SET^ mutants did not always align with the overall activity of the MLL1 complex. For example, Y3942A did not affect the MLL1^SET^ activity per se but was insufficient to support H3 methylation in the complex (Fig. 5A). Y3874A/K3878A, on the contrary, was inactive by itself but was active in the presence of Ash2L/RbBP5 (Fig. 5A). To examine the mechanism underlying the discrepancy of intrinsic versus overall H3 K4 methyltransferase activity, we decided to perform SAM binding assays for Ash2L in the context of different MLL1^SET^ mutants. To our surprise, we found a striking correlation between Ash2L SAM binding and the overall activity of the MLL1 core complex. In the MLL1 complex containing MLL1^SET^ Y3858F or Y3942A mutant, which had minimal overall activity, SAM binding by Ash2L was dramatically reduced (Fig. 5B). In contrast, SAM binding by Ash2L was only slightly affected in the complex consisting of double mutants MLL1^SET^ Y3874A/K3878A (Fig. 5B, bottom panel), which had almost no effect on overall activity (Fig. 5A, bottom panel). Duplicate samples were run for Coomassie gels as the loading controls. In the several cases we tested, SAM binding by Ash2L seemed to correlate better with the overall activity of the MLL1 complex than the intrinsic activity of MLL1^SET^. These results argue that SAM binding by Ash2L plays an indispensable role in the regulation of the overall activity of the MLL1 core complex.

### H3 and SAM mediate Ash2L and MLL1^SET^ interaction

**Figure 6:**
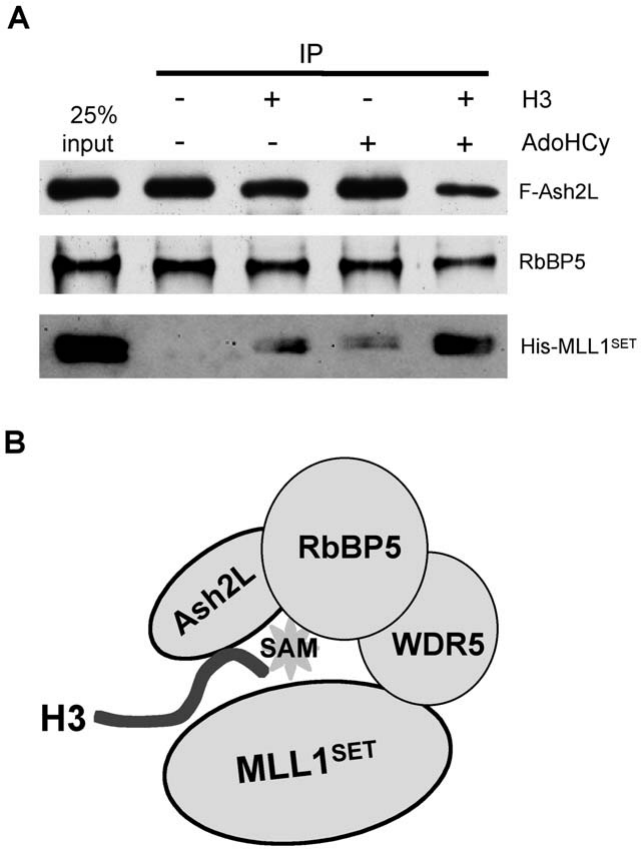
H3 and SAM mediate Ash2L/RbBP5 and MLL1^SET^ interaction. (**A**) 150 nM recombinant proteins Flag-Ash2L, His-RbBP5 and His-MLL1^SET^ were mixed in the presence of ∼7.5 µM histone H3, ∼10 µM methylation product AdoHcy or both as indicated on top. After Flag-IP, eluates from M2 agarose beads were separated on SDS-PAGE and immunoblotted for MLL1^SET^, Ash2L and RbBP5 (indicated on right). 25% input was loaded as the control. (**B**) “Single catalytic center” model for the regulation of the MLL1 core complex**.** In this model, Ash2L/RbBP5 and MLL1^SET^ interact with the same H3 substrate and substrate SAM and form a shared active center for catalysis. This structure is further stabilized by WDR5, which simultaneously interacts with RbBP5 and MLL1^SET^.

Given the highly synergistic function of Ash2L and MLL1^SET^ in H3 K4 methylation and the ability for both proteins to interact with substrate SAM, we postulate that Ash2L/RbBP5 and MLL1^SET^ may interact with the same set of H3 and SAM substrates at the catalytic center of the MLL1 complex. To test this, we performed pull-down assays for MLL1^SET^ and Ash2L/RbBP5 with or without the presence of H3 or SAM. As shown in Fig. 6, MLL1^SET^ was not able to stably interact with Ash2L and RbBP5 in the absence of WDR5, consistent with our previous observation [16]. However, in the presence of H3 or methylation product AdoHcy, MLL1^SET^ could be partially recovered by Flag-Ash2L immunoprecipitation (Fig. 6A). The pull-down efficiency for MLL1^SET^ was further enhanced when both H3 and AdoHcy were present (Fig. 6A). AdoHcy was used during the IP because Flag-IP was blocked in the presence of SAM, probably due to methylation of the M2 antibody by MLL1. Co-purification of Ash2L/RbBP5 and MLL1^SET^ in the presence of H3 and AdoHcy suggests that they probably interact with the same molecules. This result implies that Ash2L/RbBP5 probably functions at the catalytic center of the MLL1 core complex or at least in close proximity to it. The H3- and AdoHcy-dependent stabilization was only apparent in the absence of WDR5 (data not shown), which held MLL1 core complex together by simultaneously interacting with MLL1 and RbBP5 [16], [21].

## Discussion

Unlike most histone lysine methyltransferases, MLL/SET1 family H3 K4 HMTs are fully active only in the context of a multi-subunit complex and on a platform comprised of several highly conserved proteins: WDR5, RbBP5 and Ash2L [16]. Among these core components, WDR5 stably interacts with the SET domain through the residue R3765 of MLL1 [17], [20]. This interaction is critical for the integrity of the MLL1 complex [16], [17], [20]. In contrast, RbBP5 and Ash2L are not able to stably interact with MLL1 even though both proteins are capable of stimulating MLL1 methyltransferase activity [16], [22]. Here, we show that Ash2L/RbBP5 heterodimer has weak intrinsic H3 K4 methyltransferase activity (Fig. 2). This activity requires the highly conserved SPRY domain of Ash2L, as well as its interaction with RbBP5 (Fig. 2). Importantly, the ability of Ash2L to interact with H3 and SAM plays an essential role in regulating the overall activity of the MLL1 complex (Fig. 4 and 5). Our results suggest that both Ash2L/RbBP5 and MLL1^SET^ are directly involved in the catalytic reaction, which is highly unique among histone lysine methyltransferases. Given the highly conserved core configuration among MLL/SET1 family HMTs and the global effects of Ash2L (both wild type and mutants) on H3 K4 methylation in cells, it will be interesting to test whether the finding in this study is applicable for the regulation of other MLL/SET1 family members, especially SET1A/B, the predominant H3 K4 methyltransferases in mammalian cells [31].

Finding the intrinsic methyltransferase activity for Ash2L/RbBP5 is surprising since neither protein contains any known catalytic domain for histone methylation. This activity is reminiscent of what has recently been reported by Patel et al [18], even though our activity is much weaker and requires weeks of exposure for detection. Domain mapping shows that the minimal domains required for methylation include the highly conserved SPRY domain at the Ash2L C-terminus and an acidic polypeptide of 26 amino acids from RbBP5. Given the size of RbBP5 peptide and Ash2L SAM binding capability (Fig. 3), it is likely that Ash2L plays a more prominent role in the catalysis while RbBP5 stabilizes the Ash2L structure. The 250 amino acid SPRY domain of Ash2L and the closely related B30.2 domain are found in a diverse group of proteins [32]. Crystal structures of the SPRY domains in different proteins show that the SPRY domain usually forms a single-domain β-sandwich fold with one or two α-helixes and is involved in protein-protein interactions [28], [29]. Comparing the known SPRY structures with those of the SET domains and Dot1, we cannot identify features reminiscent of the active sites in known histone methyltransferases. Future structure studies of Ash2L/RbBP5 in complex with SAM and/or H3 are essential to characterize the molecular mechanism for Ash2L/RbBP5 mediated catalysis with or without the presence of MLL1.

The exact mechanism for the regulation of MLL/SET1 family HMTs remains elusive despite extensive effort. Our study here provides a novel concept that catalysis of H3 K4 methylation by the MLL1 complex not only involves the well-characterized SET domain, the catalytic domain for almost all histone lysine methyltransferases, but also a direct participation of the non-SET domain protein Ash2L. The crystal structure of MLL1^SET^ lends additional support for the requirements of other MLL1 complex components for efficient catalysis [22]. Although H3 and SAM bind to two different faces of MLL1^SET^ just as in other SET domains, the lysine access channel surrounding K4 is relatively exposed even when MLL1^SET^ is in complex with H3 peptide and SAM [22]. This open conformation implies relatively unstable alignment between H3 and SAM, underlying the poor catalytic activity. Because of this unique feature, Wilson and colleagues proposed a model that the interactions among core components and MLL1^SET^ might re-order the SET-I sub-domain of MLL1^SET^ and convert it to a closed conformation with a tightly formed lysine access channel [22]. From most well-studied SET domain structures, the formation of the lysine access channel often involves insertion of aromatic residues from flexible loop regions of the post-SET domain such as Y109 in vSET [33], W318 in DIM-5 [34] and Y337 in SET7/9 [35], [36]. The aromatic residue inserts between H3 and SAM to “lock-in” optimal orientation (Fig. S3). However, such an aromatic residue is not easily identifiable in the SET-I sub-domain of MLL1^SET^. In light of our finding that Ash2L directly interacts with SAM and H3 in the MLL1 complex, another intriguing scenario is that aromatic residue(s) from Ash2L/RbBP5 may directly participate in the catalysis and facilitate the optimal alignment of substrate lysine and SAM (Figure. 6B). Notably, there are several highly conserved aromatic residues in Ash2L SPRY (Fig. S2) and RbBP5 that can potentially fulfill this function. In this model, we envision that Ash2L/RbBP5 and MLL1^SET^ may come together to form a complete catalytic center in the MLL1 complex, each providing essential features to ensure optimal alignment of substrates for catalysis. This structure can be stabilized by WDR5, which simultaneously interact with MLL1^SET^ and RbBP5 [16], [18], [21]. Several lines of evidence support this model: 1) Ash2L/RbBP5 rescues SAM binding deficiency of MLL1^SET^ Mutant H3907A (Fig. 3D); 2) SAM binding by Ash2L/RbBP5 correlates with the overall activity of the MLL1 complex (Fig. 4B, 4C and 5); and 3) Ash2L/RbBP5 and MLL1^SET^ co-immunoprecipitate in the presence of H3 and AdoHcy even when WDR5 is absent (Fig. 6A). Future structural studies of the MLL1 core complex are imperative to distinguish these different models.

In addition to the regulation of overall activity, another important aspect of MLL1 regulation, which still remains an open question in the field, is to understand the regulation of its methylation state specificity. For most lysine methyltransferase, the specificity for mono-, di-, or tri-methylation is determined by the presence of a critical tyrosine or phenylalanine residue in the lysine access channel [37]. This residue determines whether the substrate-binding pocket allows free rotation of substrate lysine for successive addition of up to three methyl-groups. It has been proposed that Y3942 of MLL1^SET^ is a key residue to limit the intrinsic activity of MLL1^SET^ to lower methylation [18]. Mutation of Y3942 to phenylalanine converts MLL1^SET^ from a mono- to a tri-methyltransferase [18]. However, we found that mutation of Y3942 failed to support overall activity of the MLL1 complex (Fig. 5A), making it an unlikely candidate to determine methylation specificity for the MLL1 complex. Therefore, the simple “F/Y switch” model may not be able to explain the regulation of the state specificity of the MLL1 complex. Novel mechanisms, which await future studies, may be involved in this process.

## Materials and Methods

### Plasmid and Expression Vectors

For bacterial expression, SUMO tagged MLL1^SET^ (Q03164), Ash2L Full length (NP_001098684.1), Ash2L SPRY and Ash2L (1-177), WDR5 (Δ23aa, NP_060058.1), RbBP5 Full length (NP_005048.2) and RbBP5 peptides fragments were cloned into pET28a vector (Strategene); Histone H3 and its mutants were cloned into PET-3d vector. For baculovirus expression, pFastBac expression vectors (Bacvector 3000, Novagen) were used. Flag-RbBP5, His-RbBP5 were cloned into EcoRI and HindIII sites of pFastBac DUAL; His-Ash2L and His- MLL1^SET^ (3754aa- 3969aa) were cloned into BamHI and XhoI sites of pFastBac HTb; Flag-Ash2L was cloned into XhoI and HindIII sites of pFastBac DUAL. For mammalian expression, Myc-tagged Ash2L was cloned into pcDNA3 vector. Mutations were introduced by PCR-based site-directed mutagenesis.

### Protein Expression and Purification

*E. coli* BL21(DE3) cells bearing expression plasmids were induced for 16 hours with 0.1 mM IPTG at 25°C and cells were harvested by centrifugation. The cell pellets were resuspended in lysis buffer (50 mM Tris-HCl pH 8.0, 50 mM NaH2PO4, 400 mM NaCl, 3 mM imidazole, 10% glycerol, 1 mM PMSF, 0.1 mg/ml lysozyme, 2 mM 2-mercaptoethanol, and home-made protease inhibitor cocktail) and subjected to sonication. The lysate was cleared by ultracentrifugation at 38,000 rpm for 1 hour and then incubated with Ni-NTA agarose beads (Qiagen) for 2 hours at 4°C. The beads were washed by lysis buffer with 10 mM imidazole for 40 beads volume and the proteins were eluted in 250 mM imidazole. The eluted proteins were digested by ULP1 protease at 4°C overnight to remove SUMO tag. The cleaved proteins were further purified by gel-filtration chromatography on Hiload Superdex 200 in buffer A (25 mM Tris-HCl pH 8.0, 150 mM NaCl). For RbBP5 protein, an extra ionic-exchange chromatography (MonoQ) was used to increase the purity. For MLL1^SET^, protein was cleaved on-column for 3-5 hours using ULP1 in high salt buffer (50 mM Tri-HCl, 500 mM NaCl, 10% Glycerol). The purified proteins were concentrated to 10–20 mg/ml and store at −80°C. For Ash2L/RbBP5 heterodimer, equal molars of purified Ash2L and RbBP5 proteins were mixed together, incubated on ice for 30 min and purified through Hiload Superdex 200 in buffer A.

For baculovirus expressed Flag-Ash2L, His-RbBP5 and His-MLL1^SET^, cell pellets were washed with 1XPBS, resuspended in lysis buffer (20 mM Tris-HCl pH 7.9, 500 mM KCL, 20% glycerol, 1 mM PMSF, and 0.05%NP40). The cells were broken by brief sonication and cell debris was removed by centrifugation at 15,000 rpm for 20 min. The proteins were purified through epitope tag as previously described [16].

For purification of recombinant histone H3 and H3 mutant, we followed the procedure described by Luger et al. [38]. In short, the inclusion body containing histone H3 was resuspended in buffers containing 6M Urea and passed Sephacryl 200 and SP column sequentially. Histone H3 eluted from SP column was dialyzed against water and lyophilized for storage.

### Co-immunoprecipitation

For co-immunoprecipitation experiment, ∼150 nM Flag-Ash2L/His-RbBP5 complex and ∼180 nM His-MLL1^SET^ were mixed together with 7.5 µM H3, 10 µM AdoHcy or both. The purification using M2 agarose beads was carried out in BC150 buffer containing 20 mM Tris-HCl pH 7.9, 150 mM KCl, 0.2 mM EDTA and 20% glycerol at 4°C for 4–6 hours and proteins were eluted from beads by excess FLAG peptide.

### Protein Identification by LC-Tandem MS

Samples were denatured in a buffer containing 20 mM HEPES buffer, pH 7.5, and 8 M Urea. Upon reduction (10 mM DTT, 37°C, 30 min) and alkylation (50 mM iodoacetamide, 37°C, 30 min) of the cysteines, proteins were digested with 500 ng of sequencing-grade modified trypsin (Promega) after reducing the urea concentration to 1.5 M. Resulting peptides were resolved on a nano-capillary reverse phase column (Picofrit column, New Objectives) using water: acetonitrile gradient system at 300 nl/min and directly introduced into an ion-trap mass spectrometer (LTQ XL, ThermoFisher). Mass spectrometer was operated in a data-dependent mode to collect MS/MS spectra on the 5 most intense ions from each full MS scan. Proteins were identified by searching the data against human IPI database (v 3.41) appended with decoy (reverse) database using X!Tandem/Trans-Porteomic Pipeline software suite. All proteins with a Protein Prophet probability score of >0.9 were considered to be positive identifications.

### *In vitro* HMT Assay

Reactions were carried out at 30°C for 1 hour in the presence of [^3^H]-SAM (S-adenosyl-L- [methyl-^3^H] methionine) as previously described [1]. 60 µM unmodified, mono-, di-, and tri-methylated H3 K4 peptides (Millipore) or 10 µM recombinant H3 was used as substrates. Molar concentration of Ash2L, RbBP5/Ash2L and MLL1^SET^ proteins for each reaction were indicated in text or figure legend. At least three independent experiments were carried out for each figure.

### SAM Binding Assay by UV Cross-linking

Binding assay was performed as previously described with small modifications [34]. Proteins were incubated with 0.5 µCi of S-adenosyl-L- [methyl-^3^H] methionine (82 ci/mmol, Amersham TRK581) in 20 µl reaction overnight at 4°C and added to 96-well plate on ice. After 30 mins exposure from a handheld UV lamp (short wave, 254 nm) at a distance of 10 cm away from samples, the proteins were separated on SDS-PAGE, stained with Coomassie, and subjected to fluorography. For most SAM binding assays, ∼3 µM of Ash2L or Ash2L/RbBP5 or MLL1^SET^ were used. Duplicate gels were run for straight Coomassie staining as loading controls. At least three independent experiments were carried out and representative results were presented. For competitive binding, 0.35 mM unlabeled SAM or ATP was incubated with proteins in the presence of [^3^H]-SAM.

### Thermal Shift assay

The thermal shift assay was carried out using Thermofluor 384 ELS system. The Thermofluor contains a heating/cooling device for temperature control and a charge-coupled device (CCD) detector for simultaneous imaging of the fluorescence changes in the wells of the microplate. Protein unfolding was examined by monitoring the fluorescence of ANS (1-anilinonaphthalene-8-sulfonic acid) as a function of temperature with Ex/Em: 475/525 nm. All samples were prepared in quadruplicates and contained proteins at 10 µM concentrations in 50 mM Tris, pH 7.5, 150 mM NaCl buffer and 50 µM ANS. To limit the evaporation the samples were covered using mineral oil. Thermal denaturation was monitored by heating the samples from 25 to 65°C. The Tm, which is the midpoint of the protein unfolding transition, is summarized in Table S1. For proteins that do not have folded tertiary structure, we observed no thermal transition and the Tm was not determined.

### RNAi Knockdown and Rescue Experiment

HeLa cells [1] were transfected with siRNA duplexes (Dharmacon) using oligofectamine (Invitrogen) according to the manufacturer's instruction. Transfection of 1 µg Myc-Ash2L plasmid was performed 24 hour after the second of two rounds of siRNA treatment. Cells were harvested 24 hour after Ash2L over-expression. Ash2L small interfering RNAs (siRNAs) targeted 5′-CCGAGTAACTAACTTATTTAA-3′ of Ash2L 3′UTR [39].

### Antibodies

Antibodies for immunoblots were obtained commercially: anti-Ash2L (Active Motif, 39099); anti-RbBP5 (Bethyl, A300-109A), anti-FLAG (Sigma, monoclonal M2 antibody, F1804); M2 agarose (Sigma, A2220), anti-His (Qiagen, Penta his antibody, 34660); anti-myc (Millipore, 05-724), anti-H3 trimethyl K4 (Abcam, ab8580), anti-H3 dimethyl K4 (Abcam, ab32356), anti-H3 mono methyl K4 (Abcam, ab8895), anti-H3 (Upstate, 05928).

## Supporting Information

Figure S1 (A) Coomassie gels for purified recombinant proteins or protein complexes from E. coli: MLL1SET, WDR5, RbBP5, Ash2L, Ash2L/RbBP5 and Ash2L-C/RbBP5-N complex. (B) The Coomassie gel for purified recombinant proteins used in Figure 3B: RbBP5, RbBP5FD/SG, Ash2L, Ash2L R343A and MLL1SET. (C) The Coomassie gel for purified MLL1SET and MLL1SET mutant proteins as indicated on top.
(1.03 MB TIF)

Figure S2 Sequence alignment of SPRY domains of Ash2L homologues in human, Drosophila, Saccharomyces cerevisiae and Tetrahymena thermophila. The conserved Argnine residues are highlighted by square (〉). Several aromatic residues were highlighted by (*).
(0.63 MB TIF)

Figure S3 Comparison of SET domain structures for DIM5 and MLL1SET. Left, DIM5 SET domain is depicted in brown and H3 is in green. SAM is locked in position by residues 281-283 on one side and W318 on the other side. Right, MLL SET domain is depicted in pink and H3 is in blue. In this non-canonical SET domain structure, SAM and H3 are not optimally aligned. Both structures were adapted from the original structure studies [1], [2].
(1.10 MB TIF)

Table S1 Table for Tm derived from the thermal melting curves for wild type Ash2L and RbBP5 proteins as well as their mutants as indicated. RbBP5 330-363 showed no fluorescence transition in the assay, suggesting the lack of secondary structure for this peptide.
(0.12 MB TIF)
