# Supplementary figures and images for: An Ash2L/RbBP5 Heterodimer Stimulates the MLL1 Methyltransferase Activity through Coordinated Substrate Interactions with the MLL1 SET Domain
Source: PLoS One. 2010 Nov 23;5(11):e14102. doi: 10.1371/journal.pone.0014102 (PMC2990719; doi:10.1371/journal.pone.0014102)

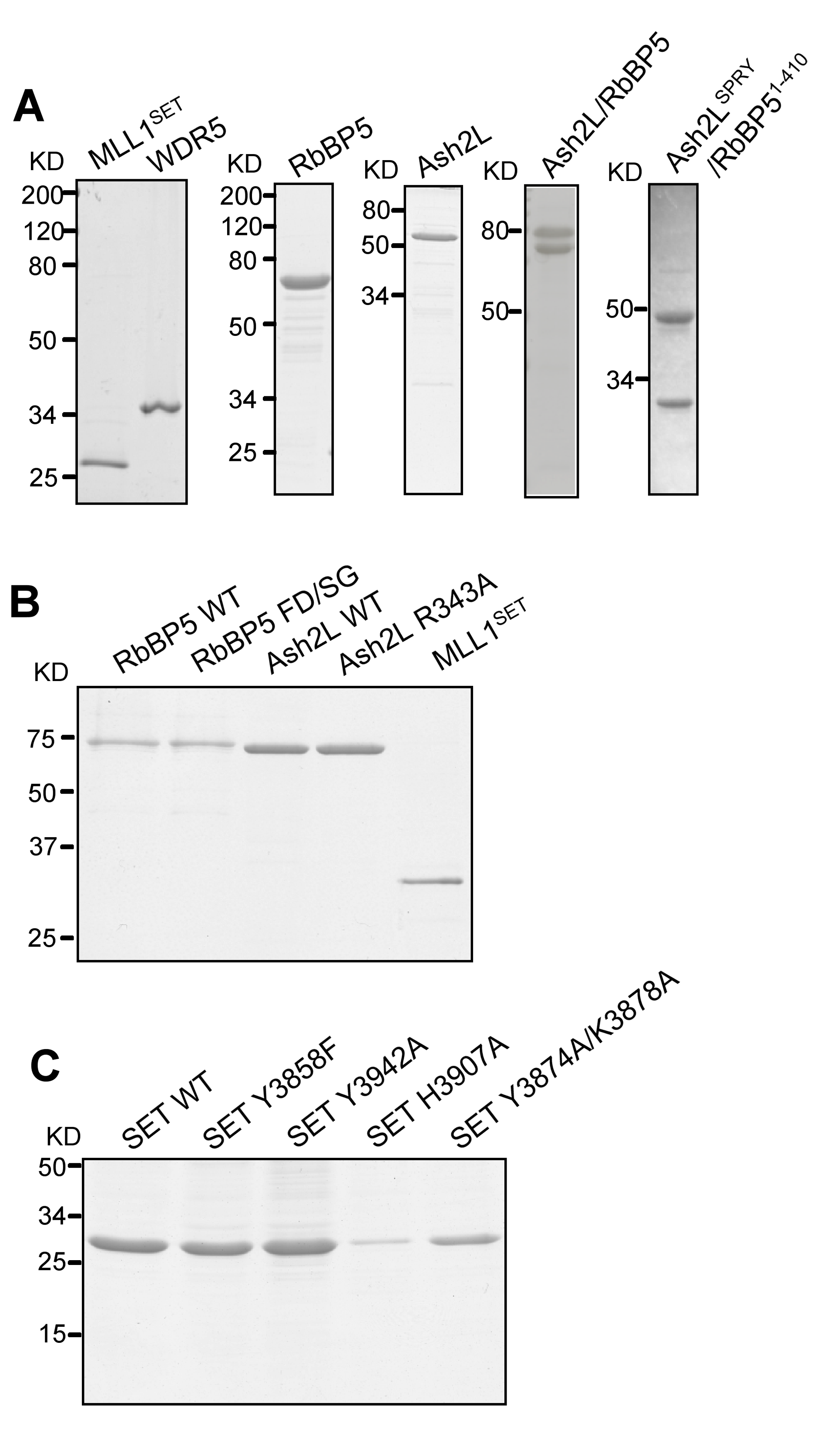

Supplement: Figure S1 — (A) Coomassie gels for purified recombinant proteins or protein complexes from E. coli: MLL1SET, WDR5, RbBP5, Ash2L, Ash2L/RbBP5 and Ash2L-C/RbBP5-N complex. (B) The Coomassie gel for purified recombinant proteins used in Figure 3B: RbBP5, RbBP5FD/SG, Ash2L, Ash2L R343A and MLL1SET. (C) The Coomassie gel for purified MLL1SET and MLL1SET mutant proteins as indicated on top. (1.03 MB TIF) [file pone.0014102.s001.tif]

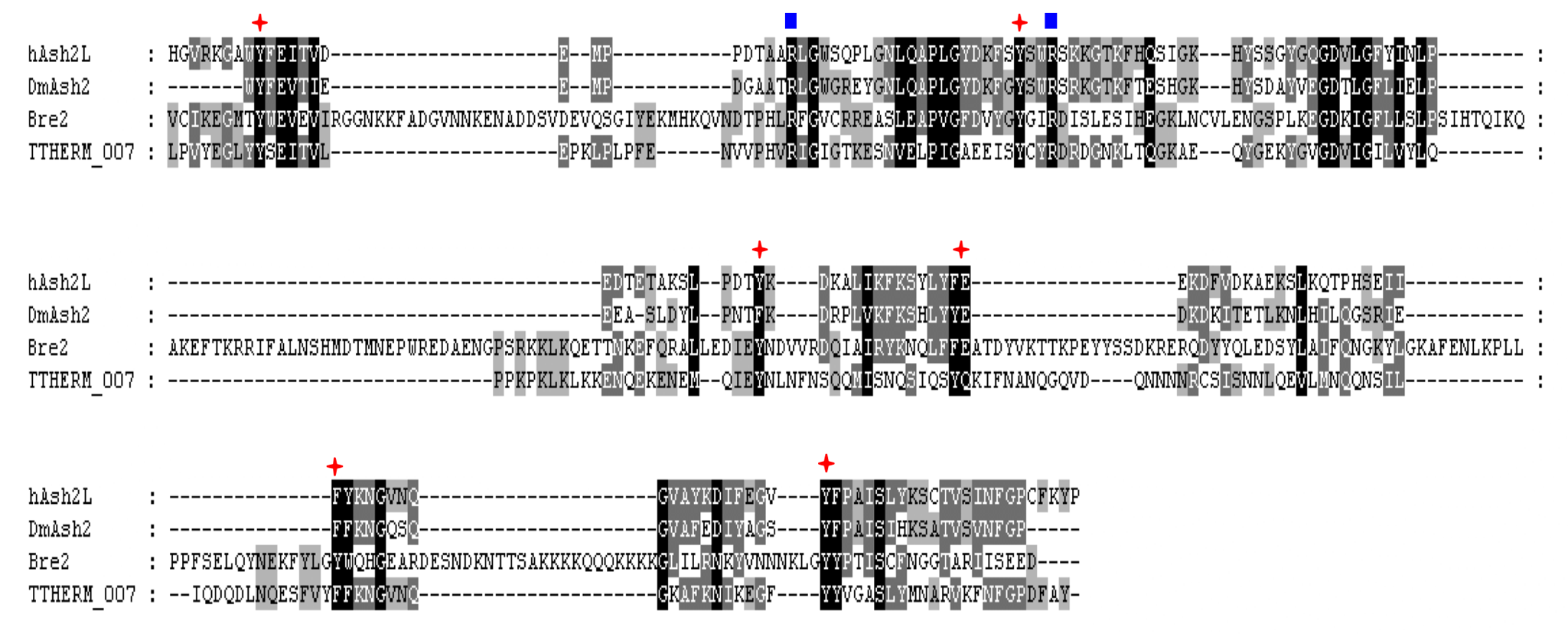

Supplement: Figure S2 — Sequence alignment of SPRY domains of Ash2L homologues in human, Drosophila, Saccharomyces cerevisiae and Tetrahymena thermophila. The conserved Argnine residues are highlighted by square (〉). Several aromatic residues were highlighted by (*). (0.63 MB TIF) [file pone.0014102.s002.tif]

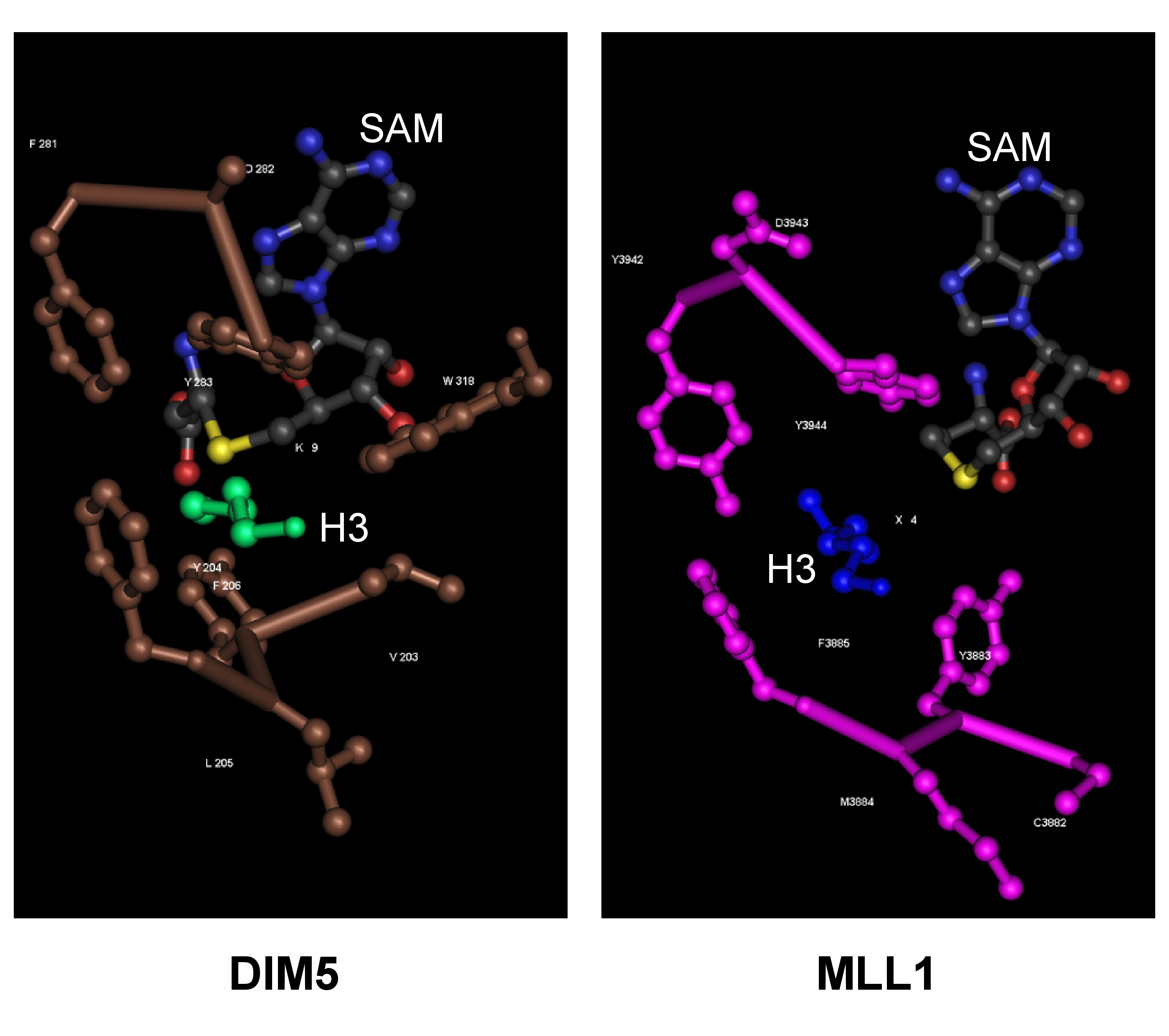

Supplement: Figure S3 — Comparison of SET domain structures for DIM5 and MLL1SET. Left, DIM5 SET domain is depicted in brown and H3 is in green. SAM is locked in position by residues 281-283 on one side and W318 on the other side. Right, MLL SET domain is depicted in pink and H3 is in blue. In this non-canonical SET domain structure, SAM and H3 are not optimally aligned. Both structures were adapted from the original structure studies [1], [2]. (1.10 MB TIF) [file pone.0014102.s003.tif]

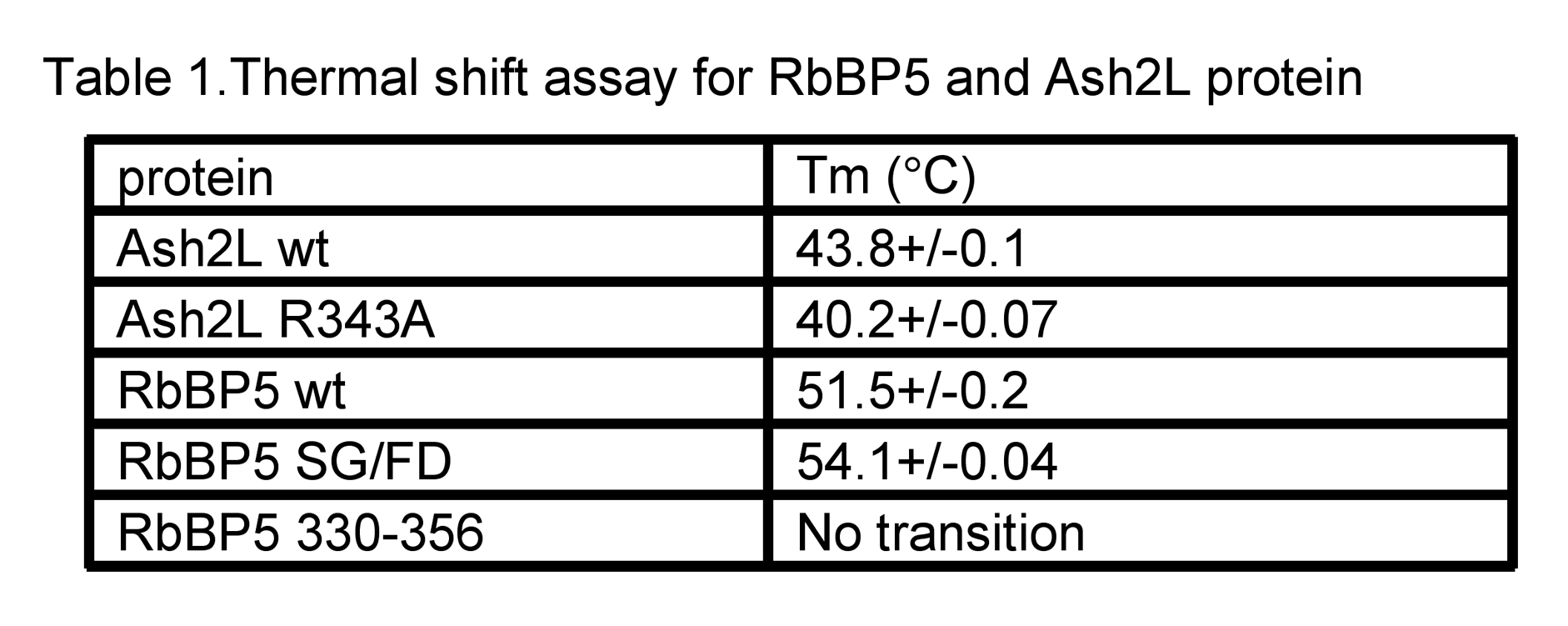

Supplement: Table S1 — Table for Tm derived from the thermal melting curves for wild type Ash2L and RbBP5 proteins as well as their mutants as indicated. RbBP5 330-363 showed no fluorescence transition in the assay, suggesting the lack of secondary structure for this peptide. (0.12 MB TIF) [file pone.0014102.s004.tif]
